# Exploring future opportunities and barriers for business model concepts in personalized nutrition

**DOI:** 10.1186/2049-3258-72-S1-K5

**Published:** 2014-06-06

**Authors:** Jo Goossens

**Affiliations:** 1 shiftN, Naamsesteenweg 255, 3001 Heverlee, Belgium

## 

Facing a growing health issue as a result if inappropriate dietary patterns, our society seems unable to convince individuals to adjust nutrition and lifestyle in an appropriate and lasting way to reduce health risks (and costs).

The sequencing of the human genome, heralded great expectations for a revolution in health care with the road to personalised health care and its extension to personalised nutrition. Although the earliest attempts fauiled to commercialise this approach, personalised nutrition, where nutrigenomics, dietary intake data, biomarker analyses and other general life-style information can be combined hold still great promises to make nutritional and dietary advice more individually acceptable and thus more effective.

Food4Me, an EU FP7 project, explores all aspects of personalized nutrition, from consumer response, scientific basis, technological tools to legal and ethical issues and value creation models. The aim is to understand what is possible, for whmo this approach could be interesting and how such an offering could succeed in the future in society.

Research based on current market offerings in this field, interviews with a wide range of stakeholders and consumer reactions to personalised nutrition concepts, shows that the key to personalised nutrition is NOT about improving nutritional advise or communicating more and better about it, it is about coaching people to bring about a lasting behavior change. THIS has more to do with behavioral science than nutrition and biology. From these analyses we built a personalised nutrition system. Dietary behaviour change sits in the center of the system and is influenced by 8 key driving forces: effectiveness of nutritional advice, economic feedback signals, force of dietary habits, psychological ambivalence (about diet and health), acceptance of genetic diagnostic information and the reliability of the risk/need profile assessment. Of course scientific excellence, especially in terms of evidence of genotype and biomarker relationships with nutrient intake, still needs to be further developed but it will to no use if the advice is not provided through good quality nutrition and lifestyle coaching or if there is no willingness to accept this. The latter will obviously require stories of positive experiences or otherwise some form of coercion.

The opportunity for novel business model concepts was then explored in 4 future scenarios about the evolution of the concept of health and the logic of health care systems in Europe.

